# Diarrhoeagenic *Escherichia coli* and *Escherichia albertii* in Brazil: pathotypes and serotypes over a 6-year period of surveillance

**DOI:** 10.1017/S0950268818002595

**Published:** 2018-09-19

**Authors:** E. L. Ori, E. H. Takagi, T. S. Andrade, B. T. Miguel, M. C. Cergole-Novella, B. E. C. Guth, R. T. Hernandes, R. C. B. Dias, S. R. S. Pinheiro, C. H. Camargo, E. C. Romero, L. F. Dos Santos

**Affiliations:** 1 Adolfo Lutz Institute-Centre of Bacteriology (National Reference Laboratory for Escherichia coli Enteric Infections), Sao Paulo, Brazil; 2 Adolfo Lutz Institute-Centre of Interdisciplinary Procedures (Culture Collections of Microorganisms), Sao Paulo, Brazil; 3 Adolfo Lutz Institute-Regional Laboratory of Santo André, Sao Paulo, Brazil; 4 Universidade Federal de São Paulo, Sao Paulo, Brazil; 5 Universidade Estadual Paulista, Botucatu, Brazil

**Keywords:** Bacterial infections, diarrhoea, *Escherichia coli* (*E. coli*), molecular epidemiology, surveillance

## Abstract

Diarrhoeagenic *Escherichia coli* (DEC) is a leading cause of infectious diarrhoea worldwide. In recent years, *Escherichia albertii* has also been implicated as a cause of human enteric diseases. This study describes the occurrence of *E. coli* pathotypes and serotypes associated with enteric illness and haemolytic uremic syndrome (HUS) isolated in Brazil from 2011 to 2016. Pathotypes isolated included enteropathogenic *E. coli* (EPEC), enteroaggregative *E. coli* (EAEC), enterotoxigenic *E. coli* (ETEC), enteroinvasive *E. coli* (EIEC) and Shiga toxin-producing *E. coli* (STEC). PCR of stool enrichments for DEC pathotypes was employed, and *E. albertii* was also sought. O:H serotyping was performed on all DEC isolates. A total of 683 DEC and 10 *E. albertii* strains were isolated from 5047 clinical samples. The frequencies of DEC pathotypes were 52.6% (359/683) for EPEC, 32.5% for EAEC, 6.3% for ETEC, 4.4% for EIEC and 4.2% for STEC. DEC strains occurred in patients from 3 months to 96 years old, but EPEC, EAEC and STEC were most prevalent among children. Both typical and atypical isolates of EPEC and EAEC were recovered and presented great serotype heterogeneity. HUS cases were only associated with STEC serotype O157:H7. Two *E. albertii* isolates belonged to serogroup O113 and one had the *stx*2f gene. The higher prevalence of atypical EPEC in relation to EAEC in community-acquired diarrhoea in Brazil suggests a shift in the trend of DEC pathotypes circulation as previously EAEC predominated. This is the first report of *E. albertii* isolation from active surveillance. These results highlight the need of continuing DEC and *E. albertii* surveillance, as a mean to detect changes in the pattern of pathotypes and serotypes circulation and provide useful information for intervention and control strategies.

## Introduction

*Escherichia coli* is as one of the most important enteric human pathogens worldwide [1]. Strains of *E. coli* causing enteric diseases are collectively designated diarrhoeagenic (DEC) and are currently divided into six main categories or pathotypes based on defined virulence attributes. The known DEC pathotypes are named enteropathogenic *E. coli* (EPEC), enteroaggregative *E. coli* (EAEC), Shiga toxin-producing *E. coli* (STEC), enterotoxigenic *E. coli* (ETEC), enteroinvasive *E. coli* (EIEC) and diffusely adherent *E. coli* (DAEC) [2].

EPEC and EAEC induce diarrhoea through their ability to adhere to host intestinal mucosa, leading to the formation of attaching and effacing (A/E) lesions in the case of EPEC and the aggregative adhesion (AA) pattern in the case of EAEC [2, 3]. Genes such as *eae* for A/E lesion and *aaf* (AA fimbriae) for AA, among others, are responsible for the production of these featured adhesion phenotypes [3, 4]. As pathogenic groups both EPEC and EAEC are subdivided in typical and atypical strains. For EPEC, this division is based upon the presence of EAF plasmid (pEAF) in typical (tEPEC) strains and its absence in atypical (aEPEC) ones [4]. The pEAF contains in its structure an operon termed *bfp*, which is responsible for the production of a type IV pilus named *bundle-forming pilus* (BFP). BFP is thought to be involved in bacteria to bacteria interactions during the host colonization by EPEC [4]. The occurrence of gene *agg*R defines typical EAEC strains while atypical EAEC are devoid of this marker [2]. Gene *agg*R is regarded as a major transcriptional regulator of many of the genes responsible for EAEC virulence factors production [3]. STEC and ETEC damage the host mainly by elaborating and secreting toxins [2]. STEC produces Shiga toxins (Stx). There are two distinct Stx types, Stx1 and Stx2 [4] with 10 subtypes, 1a, 1c and 1d for Stx1, and 2a, 2b, 2c, 2d, 2e, 2f and 2g for Stx2 [5]. ETEC produces thermolabile (LT) and thermostable (ST) enterotoxins. Both LT and ST toxins can also be divided into the distinct antigenic types LT-I and LT-II and ST-I and STII. Furthermore, ST-I may present human (ST_h_) and porcine (ST_p_) variant forms [2]. EIEC phenotypically resemble the genus *Shigella*. They are capable of invading the host intestinal mucosa and this invasive behaviour relies on a complex array of effector molecules which are employed by the bacteria in order to penetrate, evade immune response and replicate within intestinal cells [5]. The consequent inflammatory response triggered against the invasion process leads to the damage of the intestinal epithelia, characteristic of the bacillary dysentery [2].

In addition to *E. coli*, another species within the genus *Escherichia*, *E. albertii*, can also be a human pathogen. *E*. *albertii* was isolated for the first time from a diarrhoeic child in Bangladeshi and misidentified as *Hafnia alvei* [6]. Currently, *E. albertii* is considered an ‘emerging’ human enteric pathogen. Similarly to EPEC, *E. albertii* also harbours the *eae* gene and thus may produce A/E lesions. Some isolates may possess additional virulence determinants like cytolethal distending and Stx toxins [7].

EPEC, EAEC and ETEC are leading bacterial causes of acute childhood diarrhoea worldwide [8]. EPEC and EAEC have also been implicated in prolonged diarrhoeal diseases and ETEC along with EAEC are agents of the so-called ‘*traveller diarrhoea*’. On the other hand, STEC strains have been linked with large outbreaks of diarrhoea, and with the occurrence of haemorrhagic colitis and haemolytic uremic syndrome (HUS) [9].

In Brazil, the presence of DEC strains has been investigated in young children, in studies conducted at specific geographic locations [10]. However, there are no reports assessing the occurrence of DEC pathotypes from official surveillance programmes and involving patients from all age groups. Given the heterogeneous nature of DEC strains and their ability to emerge in new pathogenic forms through the gain or loss of genetic material [11], monitoring their virulence traits is of great utility as it can inform on outbreak detection. In order to provide useful epidemiologic data on the occurrence of DEC in Brazil, the present study aimed to describe the pathotypes and serotypes of *E. coli* and *E. albertii* strains associated with human infections.

## Material and methods

### Bacterial strains

The Brazilian Reference Laboratory for *E. coli* enteric infections Adolfo Lutz Institute (IAL) receives clinical isolates biochemically characterized as *E. coli* from several regional and local public health laboratories for molecular pathotype identification and serotyping. From January of 2011 to December of 2016, *E. coli* isolates representing 5047 cases of human infection, including two cases of HUS, were sent to our laboratory for this purpose. Of these, 82 cases had been previously analysed during the investigation of outbreaks of diarrhoea in the years of 2012 and 2013 [12]. These cases were also included in this study as they contribute to the total cases recorded in the period of the present study. The cases encompassed subjects of all age groups. Due to the fact that commensal *E. coli* is the predominant facultative anaerobe in the human gut, for the identification of diarrhoeagenic strains, it is necessary to evaluate more than one *E. coli*-like colony from the same patient. In our laboratory, five to 10 *E. coli* colonies from each patient are routinely received for the characterization of DEC-specific virulence markers. If more than one colony of the same pathotype is found to be positive, only one colony is considered in each case. In the present study, cases of mixed infection (two distinct DEC pathotypes occurring in the same patient) were not considered. This study also employed reference strains serving as positive controls for each of the following DEC pathotypes: EPEC (E2369/48), EAEC (17-2), ETEC (H10407), STEC/EHEC (EDL933), EIEC (*Shigella flexneri*, CDC2a). The commensal *E. coli* K12:H5 served as a negative control for molecular and phenotypic procedures.

### DEC pathotypes investigation

Screening for specific virulence genes (Table 1) defining the five most relevant DEC pathotypes (EPEC, EAEC, STEC, ETEC and EIEC) was performed by a multiplex PCR assay. For EPEC, the *eae* gene which is located in the pathogenicity island locus of enterocyte effacement (*LEE*) and is responsible for the production of the adhesin intimin was employed. For EAEC, the *aat*A gene encoding a protein related to an ATP-binding cassette transport system was used. For STEC, genes *stx*1 and *stx*2, which are bacteriophage-borne and related to the production of the Stx1 and Stx2 toxins respectively, were chosen. For ETEC, we used *lt*A and *st*A related to LT and ST toxins production, and for EIEC, the detection target was *ipa*H gene, which is associated with the invasion plasmid antigen H. Primers sequences and amplification parameters employed in the assays are described in Table 1. Template DNA for PCR reactions was produced by boiling bacterial suspensions from individual *E. coli* colonies cultivated on Tryptic Soy agar. After bacterial lysates preparation, five to 10 colonies from each patient were pooled and tested. If a given pool was positive, individual colonies forming this pool were retested with primers for the corresponding amplified gene. If a positive result was achieved, the corresponding colony was confirmed as positive.

**Table 1.** Primer sequences, target genes and amplification conditions employed in multiplex and individual PCR assays for characterizing DEC strains analysed in this study

Target gene (product/related DEC pathotype)	Primers sequences (5′–3′)	Amplification conditions	Amplicon size	Reference
*stx*1 (Shiga toxin type I/STEC)	ATAAATCGCCATTCGTTGACTAC AGAACGCCCACTGAGATCATC	95 °C 5′, 95 °C 40″, 58 °C 1′, 72 °C 2′ (40 cycles)	188	[13]
*stx*2 (Shiga toxin type II/STEC)	GGCACTGTCTGAAACTGCTCC TCGCCAGTTATCTGACATTCTG		255	
*eae* (intimin/STEC and EPEC)	GACCCGGCACAAGCATAAGC CCACCTGCAGCAACAAGAGG		384	
*ipa*H (protein associated with pINV plasmid/EIEC)	CTCGGCACGTTTTAATAGTCTGG GTGGAGAGCTGAAGTTTCTCTGC		917	
*lt*A (thermo-labile toxin/ETEC)	GGCGACAGATTATACCGTGC CGGTCTCTATATTCCCTGTT		450	
*st*A (thermostable toxin/ETEC)	ATTTTTMTTTCTGTATTRTCTT CACCCGGTACARGCAGGATT		190	
*aat*A (protein associated with an ATP-binding cassette transporter system/EAEC)	CTGGCGAAAGACTGTATCAT CAATGTATAGAAATCCGCTGTT		630	
*bfp*A (Bfp fimbriae/EPEC)	CAATGGTGCTTGCGCTTGCT GCCGCTTTATCCAACCTGGT	95 °C 5′, 94 °C 1′, 56 °C 2′, 72 °C 1′ (30 cycles)	324	
*aggR* (transcriptional virulence regulator/EAEC)	CTAATTGTACAATCGATGTA ATGAAGTAATTCTTGAAT	95 °C 5′, 94 °C 1′, 40 °C 1′, 72 °C 1′ (30 cycles)	308	[14]
*st*h (human variant of ETEC thermostable toxin/ETEC)	TTCACCTTTCCCTCAGGATG CTATTCATGCTTTCAGGACCA	95 °C 5′, 94 °C 30″, 52 °C 30″, 72 °C 1′ (35 cycles)	120	[15]
*st*p (porcine variant of ETEC thermostable toxin/ETEC)	TCTTTCCCCTCTTTTAGTCAG ACAGGCAGGATTACAACAAAG		166	

### *E. albertii* investigation

*E. albertii* was investigated by a triplex PCR assay recently described by Lindsey *et al*. This PCR targets cyclic di-GMP regulator gene (*cdg*R), DNA-binding transcriptional activator of cysteine biosynthesis gene (*EAKF1*_ch4033) and palmitoleoyl-acyl carrier protein-dependent acyltransferase gene (*EFER*_0790) allowing discrimination among *E. coli*, *E. albertii* and *E. fergusonii* [16].

### Shiga toxin genes (*stx*) subtyping

Strains presenting *stx*1 and/or *stx*2 genes were subjected to *stx* subtyping by PCR employing the primers and amplification conditions proposed by Scheutz *et al*. [17].

### Identification of typical and atypical EPEC/EAEC strains and ETEC ST toxin gene (*st*) variants

Strains presenting *eae* and *aatA* genetic markers were further investigated for *bfp* and *aggR* genes (Table 1) defining typical EPEC and EAEC, respectively. Strains negative for these genes were classified as atypical EPEC/EAEC. ST toxin gene (*st*)-positive ETEC strains were submitted to an additional duplex PCR (Table 1) in order to investigate the presence of human and porcine variants.

### Phenotypic differentiation between EIEC and *Shigella* strains

Given that *ipa*H genetic marker can be present in both EIEC and *Shigella*, and the possible occurrence of cross-reactivity among serogroups of these two bacteria in serological tests, all the strains positive for *ipa*H gene in PCR assays were submitted to an extended biochemical profiling [18]. Only strains positive for citrate, mucate and sodium acetate utilization were considered as EIEC.

### Serotyping

Strains classified in any of the DEC pathotypes investigated by PCR were O:H serotyped by tube agglutination assays [18] employing absorbed somatic (O1-O188) and flagellar antisera (H1-H56) produced at IAL. Non-motile ETEC strains of serogroup O6 were subjected to PCR-RFLP in order to identify the allelic forms of their *fli*C genes [19].

### Cytotoxicity assays

Strains harbouring *stx* genes were confirmed as STEC in cytotoxic assays employing cultured Vero cells [20].

### Statistical analyses

The *χ*^2^ test was employed to test the hypothesis that the distribution of each pathotype was not homogeneous among the distinct age groups of patients. The analysis was performed with SAS 9.3 (SAS Institute, Cary, NC). *P*-value of <0.05 was considered to indicate statistically significant differences.

## Results and discussion

DEC strains are considered major aetiological agents of diarrhoeal diseases in Brazil, and worldwide [1, 10, 21]. Nevertheless, updated information on DEC circulation in Brazilian settings is not currently available. Patterns in the circulation of diarrhoeagenic pathotypes and serotypes tend to change over time and may vary between different countries. Therefore, the primary aim of this study was to describe the occurrence of pathotypes and serotypes of DEC isolated from sporadic and outbreak cases of acute diarrhoea and HUS, during a period of 6 years of active epidemiological surveillance, performed in different Brazilian states. However, among diarrhoeagenic *eae*-harbouring *E. coli*-like colonies, we identified 10 *E. albertii* isolates, and the objective of this study was extended to encompass the analysis of such isolates.

A total of 693 (13.7%) cases were positive for DEC or *E. albertii*. DEC strains representing one of the five major pathotypes were detected as the sole enteric pathogen in 683 (13.5%) cases. *E. albertii* could be found in 10 (0.2%) of the total cases. The frequency of DEC strains reported in the present study is similar to previously reported data for China and Nigeria [22, 23], but lower than that reported in Mexico [24]. However, the real prevalence of DEC in Brazil could be greater, since in a large number of diarrhoeal cases reported, including outbreaks, the aetiologic agents are not identified due to insufficient epidemiological investigation or technical limitations. The reliable classification of DEC into distinct pathotypes requires the use of molecular tools. Since many local public health laboratories in Brazil are not adequately equipped to perform molecular techniques, most DEC infections are probably missed.

**Fig. 1.**
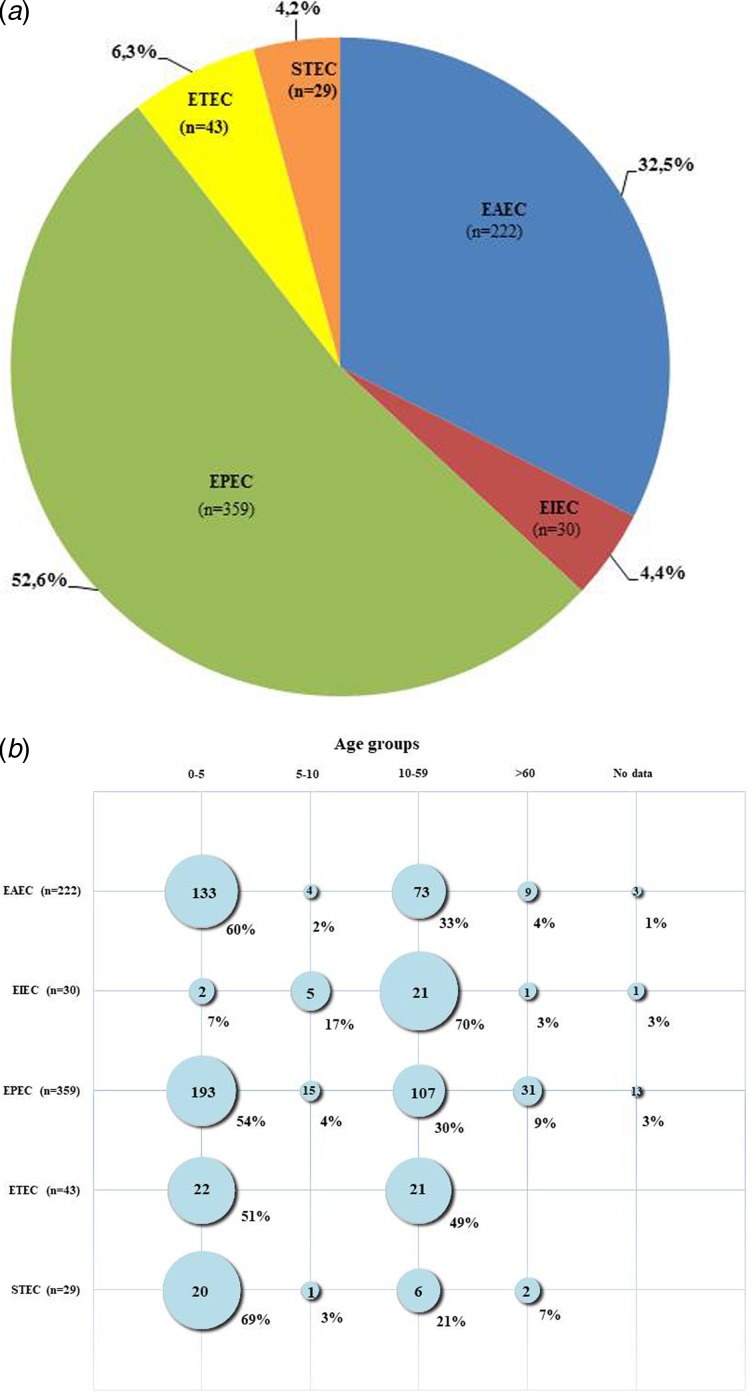
(a) Pathotypes among 683 cases of enteric infection caused by diarrhoeagenic *Escherichia coli* (DEC) in Brazil during the years of 2011–2016. (b) Occurrence of pathotypes in different age groups of patients affected by DEC strains in Brazil during the years of 2011–2016.

Figure 1a shows the distribution of the different DEC pathotypes among positive DEC strains in this study. The most frequent pathotype was EPEC, found in 359 (52.6%) of the positive DEC cases, followed by EAEC present in 222 (32.5%) of the cases. ETEC, EIEC and STEC were identified in 43 (6.3%), 30 (4.4%) and 29 (4.2%) of the positive cases, respectively. By comparing current results with studies conducted earlier in Brazil, two important differences were noticed: in prior years, EAEC strains were found to be more frequent than EPEC [10, 21], but presently, the occurrence of EPEC was higher than EAEC. In addition, according to previous reports, STEC and EIEC pathotypes were not found or were rarely diagnosed in cases of diarrhoea [10]. In this study, however, both pathotypes were found, albeit at lower frequencies compared with EPEC and EAEC. Our findings support the suggestion that there was a shift in the pattern of circulation of EAEC and EPEC strains in Brazil in recent years. Previously, EAEC were most common but EPEC have become predominant. However, the differences between the results of this study and earlier Brazilian studies may be due to the focus of earlier studies on specific regions and on children under 5 years of age [10]. So, the data they provided regarding the circulation of DEC pathotypes although useful could have been biased by local factors.

Figure 1b shows the age distribution for all DEC-positive samples collected from Brazilian patients ranging from 3 months to 96 years old. Most (370; 54%) of the DEC strains were isolated from individuals aged up to 5 years old. However, an analysis of the occurrence of the different pathotypes by age groups showed some differences regarding individual pathotypes. Most EPEC, EAEC and STEC strains were isolated from patients aged <5 years old, whereas most EIEC strains occurred among those aged >10 years old (22; 73%). ETEC infections occurred almost equally in children and adults, being found in 22 (51%) of cases involving children younger than 5 years old and in 21 (49%) cases of individuals older than 10. This pathotype did not occur in subjects older than 60. Statistical analyses demonstrated that DEC pathotypes were not equally distributed among the distinct age categories (*P* < 0.001), with the exception of ETEC, which was equally distributed between the two age groups from which this pathotype was isolated (*P* > 0.05). Enteric infections affecting young children may have serious negative consequences, so in most studies [1], including studies performed in Brazil [10, 21], this population is preferentially targeted. There is evidence that frequent and persistent infections due to DEC can lead to impairments in physical and cognitive development [25]. Moreover, age is a risk factor for HUS development after STEC infections, and children <5 years old are considered to be at greater risk [26]. The two laboratory-confirmed HUS cases in this study involved patients aged <5 years. Therefore, considering that infectious diarrhoea more often affects young children, and can also be more detrimental to them, we advise that this group of patients must receive priority for diagnosis and intervention measures.

All the identified EPEC strains of this study, except one, were classified as atypical (aEPEC), as they lacked *bfp* gene. The only typical (tEPEC) we found was a strain belonging to serotype O157:H39, isolated in 2011 from a child. Since the original description of EPEC in the middle of 1940s [27], tEPEC was the leading cause of childhood diarrhoea. However, in the 1990s, for undetermined reasons, a decline in the incidence of tEPEC was observed worldwide with concomitant rise in the incidence of aEPEC [28, 29], which is nowadays by far more prevalent than tEPEC in many locations. This trend has also been observed in Brazil [29], however, care should be taken in analysing this phenomena, as previously the identification of EPEC was based solely in serogroup determination and the presence of *bfp* gene was not routinely sought. Atypical EPEC infections affect both children and adults, and have been linked to acute, persistent and outbreaks of diarrhoeal diseases in several countries, including Brazil [12].

Among the aEPEC strains of this study, 86 serogroups were identified and their association with distinct flagellar antigens resulted in 96 different serotypes. The diversity of serogroups and serotypes found among aEPEC strains in our study is shown in Table 2. As it can be noted, no predominant serotype was identified in the period analysed, although some specific ones like O126:H19 and O33:H34 were found more often than the others. Moreover, we also observed the presence of serotypes as O145:HNM, O55:H7, O63:H6 and O26:H11/HNM that are frequently associated with STEC pathotype, raising the speculation that these aEPEC could actually represent strains that were originally STEC before loss of *stx* genes, which are bacteriophage-borne [30]. There were serotypes such as O39:HNM that have already been linked with EPEC diarrhoeal outbreaks [31]. Serotype O2:H16 in particular has been reported as an agent of aEPEC outbreaks in Brazil [12]. The finding of a great diversity of serotypes among aEPEC in this study is in agreement with other studies [32], demonstrating the heterogeneous nature of aEPEC in terms of antigenic and virulence features. It has been suggested that not all aEPEC strains are in fact pathogenic and many human subjects can be asymptomatic carriers of the bacteria [4]. Currently, we cannot determine whether all the aEPEC serotypes circulating in our settings are indeed relevant in clinical and epidemiological terms. However, it is important to continue monitoring aEPEC strains and to employ whole genome sequencing approaches to uncover the most virulent and potentially epidemic clones in order to clarify questions regarding aEPEC virulence potential.

**Table 2.** O:H antigenic types (serotypes) found among 683 DEC strains isolated between 2011 and 2016 in Brazil

DEC pathotype (total)		Serotypes (no. of isolates)
EPEC (359)		O2:H16 (6); O2:H40 (1); O2:H49 (1); O2:HNM (1); O3:H38 (2); O4:HNM (1); O7:HNM (1); O8:H10 (1); O8:H19 (1); O11:H2 (1); O11:H49 (1); O21:H21 (5); O22:H2 (1); O23:HNM (1); O25:HNM (1); O26:H11 (4); O26:H6 (1); O26:H8 (1); O26:HNM (4); O33:H34 (11); O34:H4 (2); O34:HNM (1); O35:H19 (5); O37:H45 (1); O37:HNM (3); O39:H9 (2); O43:H2 (1); O45:HNM (3); O49:H10 (6); O49:H16 (1); O49:H9 (1); O49:HNM (2); O51:H40 (4); O51:HNM (2); O55:H7 (7); O56:H6 (2); O63:H40 (1); O63:H6 (6); O63:HNM (2); O66:H21 (1); O70:H40 (2); O70:H8 (1); O71:H19 (2); O71:H49 (3); O71:HNM (2); O73:H18 (1); O76:H10 (1); O76:HNM (1); O79:H2 (1); O80:H2 (2); O81:H31 (1); O85:H4 (2); O86:H18 (1); O86:H21 (1); O86:HNM (1); O87:H10 (1); O88:H25 (5); O88:H8 (3); O88:HNM (5); O88:HNT (1); O91:H23 (3); O96:H7 (2); O98:HNM (1); O101:H33 (1); O101:HNM (1); O102:H19 (1); O103:H4 (2); O106:HNM (1); O107:H40 (1); O108:H21 (2); O108:H9 (2); O108:HNM (2); O109:H21 (6); O109:H23 (2); O109:H25 (1); O109:HNT (1); O111:H38 (1); O111:H8 (2); O112:H19 (2); O113:H19 (1); O114:HNM (3); O115:H38 (1); O117:HNM (1); O118:HNT (2); O118:HR (3); O119:H21 (1); O119:HNM (2); O121:HNM (1); O123:H19 (6); O123:HNM (2); O124:H4 (2); O125:H5 (1); O125:H6 (1); O126:H19 (12); O127:H21 (2); O127:H40 (3); O127:H45 (1); O128:H2 (2); O129:H11 (1); O131:H46 (2); O132:H34 (3); O132:H6 (1); O132:H8 (1); O133:HNM (1); O136:HNM (1); O137:H6 (3); O142:H34 (2); O144:HNM (1); O145:H34 (5); O145:HNM (7); O145:HNT (1); O145:HR (1); O146:H21 (1); O15:H1 (1); O153:H12 (1); O153:H21 (1); O153:H31 (1); O153:H7 (2); O156:H1 (3); O157:H16 (4); O157:H39^a^ (1); O157:HNM (1); O160:H14 (1); O160:H19 (3); O161:H19 (3); O162:H33 (1); O165:H9 (1); O170:H49 (1); O177:H9 (2); O177:HNM (1); O177:HNM (1); O179:H31 (1); O180:HNM (3); O181:H18 (1); O181:HNM (1); ONT:H10 (2); ONT:H11 (1); ONT:H19 (5); ONT:H2 (1); ONT:H21 (7); ONT:H23 (1); ONT:H30 (1); ONT:H4 (3); ONT:H40 (4); ONT:H43 (1); ONT:H49 (2); ONT:H5 (1); ONT:H6 (5); ONT:H8 (1); ONT:H9 (1); ONT:HNM (7); ONT:HNT (5); ONT:HR (1); OR:H1 (1); OR:H10 (2); OR:H16 (2); OR:H19 (5); OR:H21 (4); OR:H23 (1); OR:H25 (1); OR:H31 (1); OR:H33 (3); OR:H4 (2); OR:H40 (1); OR:H49 (1); OR:HNM (2); OR:HR (1)
EAEC	Typical (187)	O3:H2 (3); O5:H10 (2); O9:H10 (3); O11:H18 (1); O15:H18 (4); O15:H2 (9); O20:H30 (1); O21:H2 (4); O25:H4 (1); O38:H25 (2); O39:H9 (1); O44:H18 (2); O55:H21 (1); O59:H19 (2); O59:HNM (2); O68:H1 (1); O73:H1 (4); O73:H18 (4); O73:H33 (1); O73:HNM (1); O82:H10 (1); O84:HNM (1); O86:H2 (4); O99:H4 (1); O99:H6 (3); O104:H4 (3); O106:H18 (1); O106:HNM (1); O114:H10 (1); O131:H25 (1); O138:H48 (1); O153:H2 (9); O153:H28 (1); O153:HNM (2); O155:H19 (2); O168:HNM (4); O175:H23 (1); O175:H28 (11); O176:H33 (5); O176:H34 (1); O179:HNM (1); O181:H28 (2); ONT:H1 (2); ONT:H10 (16); ONT:H18 (3); ONT:H2 (2); ONT:H21 (2); ONT:H33 (1); ONT:H4 (2); ONT:H9 (1); ONT:HNM (9); OR:H1 (1); OR:H10 (2); OR:H17 (1); OR:H18 (7); OR:H2 (10); OR:H21 (1); OR:H25 (2); OR:H33 (1); OR:H4 (2); OR:H6 (1); OR:HNM (15)
	Atypical (35)	O11:H18 (1); O15:H2 (1); O38:H25 (1); O43:H2 (1); O55:H25 (2); O70:H8 (1); O73:H18 (1); O80:H10 (3); O81:H27 (1); O139:H19 (1); O170:H49 (1); O175:H28 (1); ONT:H32 (2); ONT:H33 (1); ONT:HNM (2); OR:H10 (3); OR:H2 (1); OR:H21 (1); OR:H25 (2); OR:H33 (3); OR:H35 (2); OR:H45 (1); OR:HNM (2)
STEC (29)		O8:H19 (1); O24:H4 (1); O26:H11 (1); O71:H8 (1); O91:H14 (1); O100:HNM (1); O103:HNM (1); O111:H11 (1); O111:H8 (2); O111:HNM (4); O118:H16 (1); O123:H2 (1); O123:HNM (3); O145:HNM (1); O153:H21 (1); O153:H7 (1); O157:H7 (2); O177:HNM (1); O178:H19 (1);ONT:H19 (1); ONT:H46 (1); OR:HNM (1)
ETEC (43)		O6:H16 (22); O6:HNM (3); O25:HNM (6); O64:H21 (1);O109:H19 (1); O159:H17 (1); O159:H21 (1); O165:H15 (1); O166:H15 (2); ONT:H10 (1); ONT:H2 (1); ONT:H8 (1); ONT:HNM (1); OR:H31 (1)
EIEC (30)		O2:HNM (1); O18:H31 (1); O121:HNM (7); O124:H30 (4); O124:HNM (2); O132:H21 (8); O132:HNM (1); O135:HNM (1); O144:HNM (3); ONT:HNM (2)

OR, O rough; ONT, O non-typeable; HNM, H non-motile; HNT, H non-typeable.

a Typical EPEC (*bfp+*).

In our analysis, strains carrying *agg*R gene, thus classified as typical EAEC, were 84% (187/222) of the total of EAEC-positive strains, while 16% (35/222) of the EAEC in this study were atypical and lacked the gene. These findings are similar to previous reports that the majority of the EAEC strains linked to enteric illness harbour *agg*R [33]. As the *agg*R gene product regulates the expression of most of the currently identified virulence factors of EAEC, in some studies, it is suggested that only typical EAEC are pathogenic for humans [3]. However, there has been a report implicating atypical EAEC with diarrhoeal cases [34]. The fact that in this study we did not find any other bacterial or viral enteropathogens in samples positive for atypical EAEC corroborates this previous report and is evidence for a role for atypical EAEC in enteric illness.

EAEC strains in this study included 42 distinct O:H serotypes, 35 of them associated with typical isolates and 12 associated with atypical EAEC (Table 2). Serotypes O11:H18, O15:H2, O175:H28, O38:H25, O73:H18 were common to both typical and atypical strains, and one can speculate that these aEAEC belonging to these serotypes of typical EAEC could have lost *agg*R-bearing plasmid. Serotypes O175:H28, O15:H2 and O153:H2 were more commonly found among typical EAEC. However, the majority of the EAEC strains of this study, irrespective of the fact they were typical or atypical, could not have their O antigens determined due to roughness or absence of antisera reactivity. The self-agglutinating nature of EAEC strains together with serotype diversity [35, 36] limits the use of serological techniques in outbreaks tracing and laboratory surveillance. Nevertheless, some interesting findings regarding antigenic features of EAEC of this study could be observed. The H2 antigen was frequently associated with typical EAEC. Some of the serotypes found in this study have been reported in other studies conducted in Brazil, but serotypes O175:H28 and O15:H2 were not previously reported, though they were the most common serotypes found in the period of this study [37, 38]. Strains of serogroup O15 with H18 antigen had been reported previously in Brazil as atypical EAEC [37]. In this study, O15 serogroup was found in combination with H2 flagellar type and mostly among typical EAEC.

ETEC is a major cause of moderate-to-severe diarrhoea in developing countries especially in Asia [39].This pathotype is also an important enteric pathogen in South America and earlier studies in Brazil have reported ETEC infections, including outbreaks, in different regions [10, 21, 40]. In the present study, ETEC was isolated in 43 (6.3%) of the total of diarrhoeal cases analysed. Our results confirm that although less frequent than other DEC pathotypes such as EPEC and EAEC, ETEC are still responsible for causing enteric illness in our country, and must therefore continue to be considered in the list of enteric pathogens to be sought for the diagnosis of diarrhoeal diseases. In this study, we found the profile *lt*+/*st*+ as the most common toxigenic genotype among ETEC-positive isolates, being present in 56% (24/43) of these strains, while 44% (19/43) of the strains harboured only LT enterotoxin-related gene *lt*. None of the strains studied carried *st* gene alone. It has been reported that *st* or *st*/*lt* carrying ETEC strains, rather than *lt* only harbouring ETEC, are more often associated with moderate-to-severe diarrhoea and a higher risk of death in young children [1]. In the *st*+ isolates, *st*_h_ gene variant was carried by 21 strains, while only one strain possessed the *st*_p_ variant. ST enterotoxin variants STh and STp can both induce diarrhoea in humans; however, the human variant is considered to be more relevant in clinical terms due to its higher prevalence when compared with STp [41]. Three ETEC strains did not give any result in relation to the *st* gene variants searched. No information regarding the toxigenic profiles of ETEC isolated previously in Brazil is available; therefore, our findings although derived from a small number of strains are the only data available on this topic. Examination of the 43 ETEC isolates for O:H antigens demonstrated the occurrence of 14 distinct serotypes (Table 2). However, 25 (58%) of these strains belonged to the single serotype O6:H16, including 22 motile strains (H16 antigens expressed) and three non-motile strains (H16 type was determined by PCR-RFPL analysis of *fli*C genes). ETEC of serotype O6:H16 is of worldwide occurrence, being one of the most common serotypes associated with ETEC infections in humans [2].

All the strains positive for *stx* genes in this study were phenotypically confirmed as STEC in Vero cell cytotoxic assays. We had 19 strains (65.5%) carrying *stx*1, while nine (31%) carried only *stx*2 and one strain carried both *stx*1 and *stx*2 (Table 3). Previous studies characterizing STEC of clinical origin in Brazil have also reported that most of the isolates harboured only *stx*1 and were from cases of acute non-complicated diarrhoea [42]. Subtyping of *stx* genes revealed that *stx*1a allele was carried by all the *stx*1-positive strains, except one that had *stx*1d (Table 3). In relation to stx2 subtypes, we encountered the allele 2a in association with 2c, 2d or 2e in five of the *stx*2-positive STEC, while subtypes 2c and 2e were found alone in four strains (Table 3). Subtypes 2b and 2g were not present. Stx2 and subtypes 2a, 2c and 2d are more often linked with complicated STEC infections and their association with some specific O serogroups and adherence factors can be a predictor of greater probability of HUS [43–45]. In this study, we were able to demonstrate that these three most problematic *stx*2 subtypes were found in most of *stx*2-positive strains. However, STEC strains producing Stx1 can also cause HUS [45], so the possibility that *stx*1a-bearing Brazilian isolates can be responsible for more complicated infections exist and for this reason they must be carefully monitored.

**Table 3.** Serotypes and *stx* genotypes among 29 STEC strains recovered from human infections in Brazil from 2011 to 2016

Strain	Year of isolation	Serotype	*stx* genotype	Presence of *eae*	Clinical condition
179/11	2011	OR:HNM	2c	+	AD
61/12	2012	O100:HNM	2e	−	AD
75/12		O111:HNM	1a,2a	+	AD
169/12		O177:HNM	2c	+	AD
343/12		O153:H21	1a	−	AD
359/12		O111:H8	1a	+	AD
611/12		O111:HNM	1a	+	AD
340/13	2013	O157:H7	2a,2c	+	HUS
377/13		O24:H4	1a	−	AD
423/13		O118:H16	1a	+	AD
444/13		O103:HNM	1a	+	AD
502/13		O111:H8	1a	+	AD
605/13		O71:H8	1a	+	AD
438/14	2014	O111:H11	1a	+	AD
504/14		O145:HNM	1a	+	AD
544/14		O153:H7	1d	−	AD
579/14		O123:HNM	1a	+	AD
589/14		O91:H14	1a	−	AD
672/14		O8:H19	2a,2d	−	AD
P059-9/14		026:H11	1a	+	BD
302/15	2015	ONT:H46	2a,2d	−	AD
516/15		O123:H2	1a	+	AD
768/15		O111:HNM	1a	+	AD
831/15		O157:H7	2a,2c	+	HUS
927/15		O123:HNM	1a	+	AD
P001/15		ONT:H19	2a, 2e	−	AD
254/16	2016	O123:HNM	1a	+	AD
583/16		O178:H19	2c	−	AD
811/16		O111:HNM	1a	+	AD

AD, acute diarrhoea; BD, bloody diarrhoea; HUS, haemolytic uremic syndrome.

Twenty (69%) of the STEC strains herein analysed possessed *eae* gene, while nine strains (31%) lacked this marker (Table 3). This indicates that the majority of the human STEC infections in Brazil, in the period covering this study, were caused by strains dotted with the potential ability to express both Shiga toxin and A/E phenotypes. The potential to form A/E lesions by STEC isolates is regarded as an additional risk factor in the clinical outcome of STEC diseases, as there is a higher risk of HUS development [9]. In fact, most of the HUS cases registered in Brazil [46] including the two cases analysed in this study were caused by strains carrying *stx*2 and *eae*. In this study, STEC strains fell into 15 distinct serotypes (Table 2), and included serotypes of major epidemiological importance such as O157:H7, O111:H8/NM, O26:H11, O145:HNM, O103:HNM, as well as serotypes which have been implicated in human disease, but isolated less frequently [47]. The most frequent serotype presently observed was O111:H8. The same situation was observed in prior years in Brazil where STEC O111 was the most frequent serogroup encountered in human diseases [42]. By comparing the present results with data about STEC serotypes in Brazil spanning the period of 1979–2004, we could note that the diversity of serotypes detected in this study was greater than that observed before. This may be indicative of the efforts that have been made in Brazil to increase the detection of STEC pathogens by employing molecular approaches targeting *stx* genes, instead of serogroup-based screening by immunological methods, which were largely performed in the past. This change in methodology must continue and should be implemented in the largest possible number of laboratories in Brazil, for the benefit of future surveys addressing STEC infection epidemiology.

Studies describing the occurrence and markers for EIEC circulation are scarce. This is due to the fact that EIEC differentiation from *Shigella* is often problematic as these two bacteria are closely related and almost identical in terms of genetic contents. Additionally, surface antigens of EIEC and *Shigella* cross-react, so serological tests may not be a suitable option. In fact, there is evidence from phylogenetic studies demonstrating that EIEC strains represent intermediate forms in the evolution from commensal *E. coli* to *Shigella* [48]. Differentiation among EIEC and *Shigella* is possible only through extensive biochemical profiling which can be performed solely in reference laboratories. As a consequence, EIEC strains are under-represented in most epidemiological surveys. Therefore, EIEC contribution to the burden of DEC infections is largely overlooked. In this study, phenotypically confirmed EIEC corresponded to 4.4% of DEC strains, showing that this pathotype has a role in community-acquired diarrhoea in Brazil. Serogroups O132, O121 and O124 were the most common, being present in 9/30 (30%), 7/30 (23%) and 6/30 (20%) of strains, respectively, and serotype O132:H21 was the most frequent. Serogroups O121 and O124 are among the most commonly reported among EIEC strains [2], and in this sense, our results only partially agree with previous reports, as in this study the O132 serogroup was the most prevalent. EIEC outbreaks have been reported in other countries [49] and we are currently performing PFGE typing to assess the genetic relatedness among strains of the same serotype isolated in this study.

Ten of the *eae*-harbouring strains, which had been previously identified in our routine laboratory testing as EPEC, were actually found to be *E. albertii*. The recognition of *E. albertii* is challenging in that with few exceptions their biochemical profile and most of the virulence markers resemble DEC pathotypes EPEC and STEC. Only recently genomic approaches have allowed accurate discrimination, reallocating these strains to another taxonomic position [50]. This is the first report on the occurrence of *E. albertii* from active surveillance of foodborne diseases in Brazil. The majority of the *E. albertii* were untypeable or rough regarding their somatic antigens, and were non-motile, so their O:H serotypes could not be identified. This is in agreement with previous reports of the antigenic untypeability of *E. albertii* strains [51]. It is worth mentioning that there is no specific typing scheme for *E. albertii* and attempts to determine their somatic and flagellar antigens usually employ antisera produced against *E. coli* strains. This suggests that *E. albertii* O and H antigens may have distinct characteristics in relation to *E. coli* antigens. However, there were two exceptions in this study, as two strains reacted with O113 *E. coli* antisera, but were non-motile, rendering serotype O113:HNM. One of the analysed *E. albertii* strains in this study was positive for *stx*2f gene. Production of Stx2f by *E. albertii* strains has been reported [7] and one can speculate about the potential of these strains to cause more serious diseases. So far, *E. albertii* appears to represent a small proportion of the diarrhoeagenic strains circulating in Brazilian settings, but even so they must receive attention in surveillance programmes in Brazil and elsewhere, so that it will be possible to determine how their circulation trends will evolve.

We attempted to draw a scenario for DEC strains occurrence in comparison to prior years in Brazil, and with data from other countries. Although several problems were faced, especially related to logistical difficulties in sending bacterial isolates to reference laboratories for analysis, we believe the present study contributes a useful ‘*snapshot*’ on the aetiology of diarrhoeal diseases caused by DEC strains in our country. Certainly this will be very important for future studies and considering intervention measures. The continuous epidemiological surveillance of food and water transmissible diseases and characterization of DEC strains associated with human infections is essential for the recognition of new patterns of pathogen virulence and circulation. In this regard, it is of paramount importance that public health and clinical laboratories involved in infectious diseases diagnosis and surveillance are capable of correctly recognizing DEC strains.
